# Partial restoration of spinal cord neural continuity via vascular pedicle hemisected spinal cord transplantation using spinal cord fusion technique

**DOI:** 10.1111/cns.13853

**Published:** 2022-05-12

**Authors:** Xiaoping Ren, Weihua Zhang, Jie Qin, Jian Mo, Yi Chen, Jie Han, Xinjian Feng, Sitan Feng, Haibo Liang, Liangjue Cen, Xiaofei Wu, Linxuan Han, Rongyu Lan, Haixuan Deng, Huihui Yao, Zhongquan Qi, Hongjun Gao, Lishan Wei, Shuai Ren

**Affiliations:** ^1^ Department of Orthopedics Ruikang Hospital Affiliated to Guangxi University of Chinese Medicine Nanning China; ^2^ Institute of Orthopedics Ruikang Hospital Affiliated to Guangxi University of Chinese Medicine Nanning China; ^3^ Global Initiative to Cure Paralysis (GICUP) Columbus Ohio USA; ^4^ Department of Imaging Ruikang Hospital Affiliated to Guangxi University of Chinese Medicine Nanning China; ^5^ Department of Electrophysiology Ruikang Hospital Affiliated to Guangxi University of Chinese Medicine Nanning China; ^6^ 12664 Medical College Guangxi University Nanning China; ^7^ Department of Organ Transplantation Ruikang Hospital Affiliated to Guangxi University of Chinese Medicine Nanning China; ^8^ Department of Orthopedics the Second Affiliated Hospital of Harbin Medical University Harbin China

**Keywords:** clinic trial, GEMINI, polyethylene glycol, spinal cord fusion, spinal cord injury

## Abstract

**Aims:**

Our team tested spinal cord fusion (SCF) using the neuroprotective agent polyethylene glycol (PEG) in different animal (mice, rats, and beagles) models with complete spinal cord transection. To further explore the application of SCF for the treatment of paraplegic patients, we developed a new clinical procedure for SCF called vascular pedicle hemisected spinal cord transplantation (vSCT) and tested this procedure in eight paraplegic participants.

**Methods:**

Eight paraplegic participants (American Spinal Injury Association, ASIA: A) were enrolled and treated with vSCT (PEG was applied to the sites of spinal cord transplantation). Pre‐ and postoperative pain intensities, neurologic assessments, electrophysiologic monitoring, and neuroimaging examinations were recorded.

**Results:**

Of the eight paraplegic participants who completed vSCT, objective improvements occurred in motor function for one participant, in electrophysiologic motor‐evoked potentials for another participant, in re‐establishment of white matter continuity in three participants, in autonomic nerve function in seven participants, and in symptoms of cord central pain for seven participants.

**Conclusions:**

The postoperative recovery of paraplegic participants demonstrated the clinical feasibility and efficacy of vSCT in re‐establishing the continuity of spinal nerve fibers. vSCT could provide the anatomic, morphologic, and histologic foundations to potentially restore the motor, sensory, and autonomic nervous functions in paraplegic patients. More future clinical trials are warranted.

## Graphical abstract

In this clinical trial, vascular pedicle hemisected spinal cord transplantation (vSCT), one of the spinal cord fusion clinical translation models, was demonstrated the clinical feasibility and efficacy in re‐establishing the continuity of spinal nerve fibers. vSCT could provide the anatomic, morphologic, and histologic foundations to potentially restore the motor, sensory, and autonomic nervous functions in paraplegic patients.

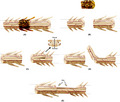

## 1 INTRODUCTION

Spinal cord injury (SCI) is a disease with a high morbidity and mortality rate. It is estimated that 27 million people worldwide are paralyzed by SCI, and 500,000 new cases occur each year. Patients with paraplegia are confined to a wheelchair and subjected to a lifetime of multiple medical comorbidities.^1^ In addition to greatly reducing the quality of life of patients, paraplegia also brings a huge social burden due to high medical costs and loss of productivity in the workplace.^2^ Therefore, treatment to restore motor function in these patients would be a great advance.

In our proposed neurologic foundations of spinal cord fusion (SCF), which is called GEMINI, polyethylene glycol (PEG) was used as a neuroprotective fusogen to fuse two approximated stumps of the transected spinal cord.^3^, ^4^, ^5^ PEG is an inexpensive, stable, nontoxic, fully biocompatible, and water‐soluble linear polymer that is synthesized by the living anionic ring‐opening polymerization of ethylene oxide and has molecular weights ranging from 0.4 to 100 kDa.^6^ PEG is known to promote the fusion of plant protoplasts.^7^ In 1975, Pontecorvo reported that PEG could be used as a fusogen to fuse mammalian cells to produce hybrid cells.^7^ In 1986, Bittner et al.^8^ showed that PEG has the ability to fuse and reseal the membranes of severed axon membranes in vitro under controlled conditions. Subsequently, in 1999, Shi et al^9^ demonstrated that PEG could fuse completely transected strips of isolated white matter in vitro. These studies suggest that PEG might be used to reconstruct not only peripheral nerves but also severed spinal cord axons in vivo. More recently, a number of molecular agents have been tested in different models of SCI; they include astaxanthin,^10^ and Chitosan combined therapy.^11^

Since 2016, our team has found that PEG could restore the motor function of hind limbs in different animal models of paraplegia (complete spinal cord transection at T10), such that mice and rats regained the ability to stand and crawl using their hind legs about 1 month after surgery,^12^, ^13^ and beagles regained the ability to stand and crawl about 2 months after surgery.^14^, ^15^

In addition to PEG, the cortico‑trunco‑reticulo‑propriospinal (CTRPS) pathway mentioned in GEMINI also plays an important role in SCF.^3^, ^4^, ^5^ The CTRPS is the phylogenetically oldest motor and sensory command system. As the species evolved, the pyramidal tract, a faster command system, developed allowing for rapid transmission of volitional signals. After spinal cord transection, however, even if no pyramidal tract axon could be fused by PEG. The propriospinal neurons of the CTRPS along with others in proximity that were not damaged by the extra‐sharp blade used to transect the spinal cord can regrow (sprout) their fibers immediately and re‐establish contacts between the apposed interfaces.^3^, ^5^ The entire motor and sensory recovery would thus hinge on the CTRPS pathway.^16^, ^17^

In the GEMINI and our previous animal experiments, paraplegic models were constructed by quickly and acutely transecting the spinal cord with an extremely sharp surgical blade that imparted minimal local trauma. There was no gap between the adjacent spinal cord stumps. However, clinical paraplegic patients often have extensive spinal cord contusion and fibrous scarring, with numerous cysts and fibrous connective tissue response in the area of SCI. Therefore, we developed several clinical translation models of SCF for paraplegic patients and conducted a clinical trial (http://www.chictr.org.cn/showproj.aspx?proj=50526, ChiCTR2000030788) of SCF. In this report, we focus on one of the SCF clinical translation models: vascular pedicle hemisected spinal cord transplantation (vSCT). Prior to this clinical trials, we used beagle dogs as animal models to verify the feasibility of vSCT and the efficacy of PEG‐600 (see Appendix [Link], [Link], [Link], [Link]). Herein, we present the preliminary postoperative results of eight participants treated with vSCT in the clinic. This paper demonstrates that vSCT, a clinical translational model of SCF, appears to be a potential therapeutic approach in the field of SCI.

## 2 METHODS

### 2.1 SCF clinical trial

This clinical trial was approved by the Medical Ethics Committee of Ruikang Hospital Affiliated to Guangxi University of Traditional Chinese Medicine and registered on the open clinical registry site http://www.chictr.org.cn/showproj.aspx?proj=50526 (ChiCTR2000030788). All treatment operations in this clinical trial followed the ethical standards of the hospital and the 1975 Declaration of Helsinki and its subsequent revisions and similar ethical standards. A formal informed consent form was developed and approved by the Medical Ethics Committee of Ruikang Hospital Affiliated with the Guangxi University of Traditional Chinese Medicine. The study design of this clinical trial was a single‐arm trial.

### 2.2 Participant selection and evaluation

Our inclusion criteria were 1) age ≤50 years old; 2) a traumatic‐chronic or acute‐complete type of SCI; 3) SCI located in the thoracic segment; 4) able to understand and complete the trial; and 5) normal cardiopulmonary function to complete the prolonged operation (ASA:1–2).^18^

Our exclusion criteria were 1) participant age >50 years old; 2) incomplete type of SCI or pathological SCI; 3) SCI located in the cervical and lumbar segments; 4) inability to cooperate with doctors for the trial, such as due to mental disorders or associated traumatic brain injury; and 5) poor cardiopulmonary function that would prevent completion of the prolonged operation (ASA:3–6).^18^

If the participants met these requirements, informed consent was obtained. During the obtaining of informed consent, which was filmed with participant consent, we addressed the surgical risks, complications, and prognosis of the SCF. Paraplegic participants often had injuries with spinal fractures and were previously treated with spinal canal decompression and spinal stabilization at their local hospitals. Their spinal hardware of thoracic or lumbar spine stabilizations were removed before our clinical trial. MRI, diffusion tensor imaging (DTI),^19^, ^20^, ^21^ computerized tomography (CT), somatosensory‐evoked potential (SSEP), and motor‐evoked potential (MEP) were performed to assess SCI and spinal fracture.

### 2.3 Preoperative preparation for surgery

Before surgery, the surgical team discussed the SCI condition with the participants. Participants completed routine examinations before the surgery, including laboratory examination, electrocardiogram, and ultrasonography. Participants stopped smoking 2 weeks before the operation. Tilopidine and clopidogrel, aspirin, and non‐steroidal anti‐inflammatory drugs were discontinued 10, 7, and 2 days before the operation, respectively. Conditions, such as malnutrition, hypertension, and high blood sugar were addressed and controlled before the surgery. Prophylactic antibiotics were administered 30 min before the surgery. Tranexamic acid was administered to minimize bleeding during the surgery.

### 2.4 Vascular pedicle hemisected spinal cord transplantation (vSCT)

**FIGURE 1:**
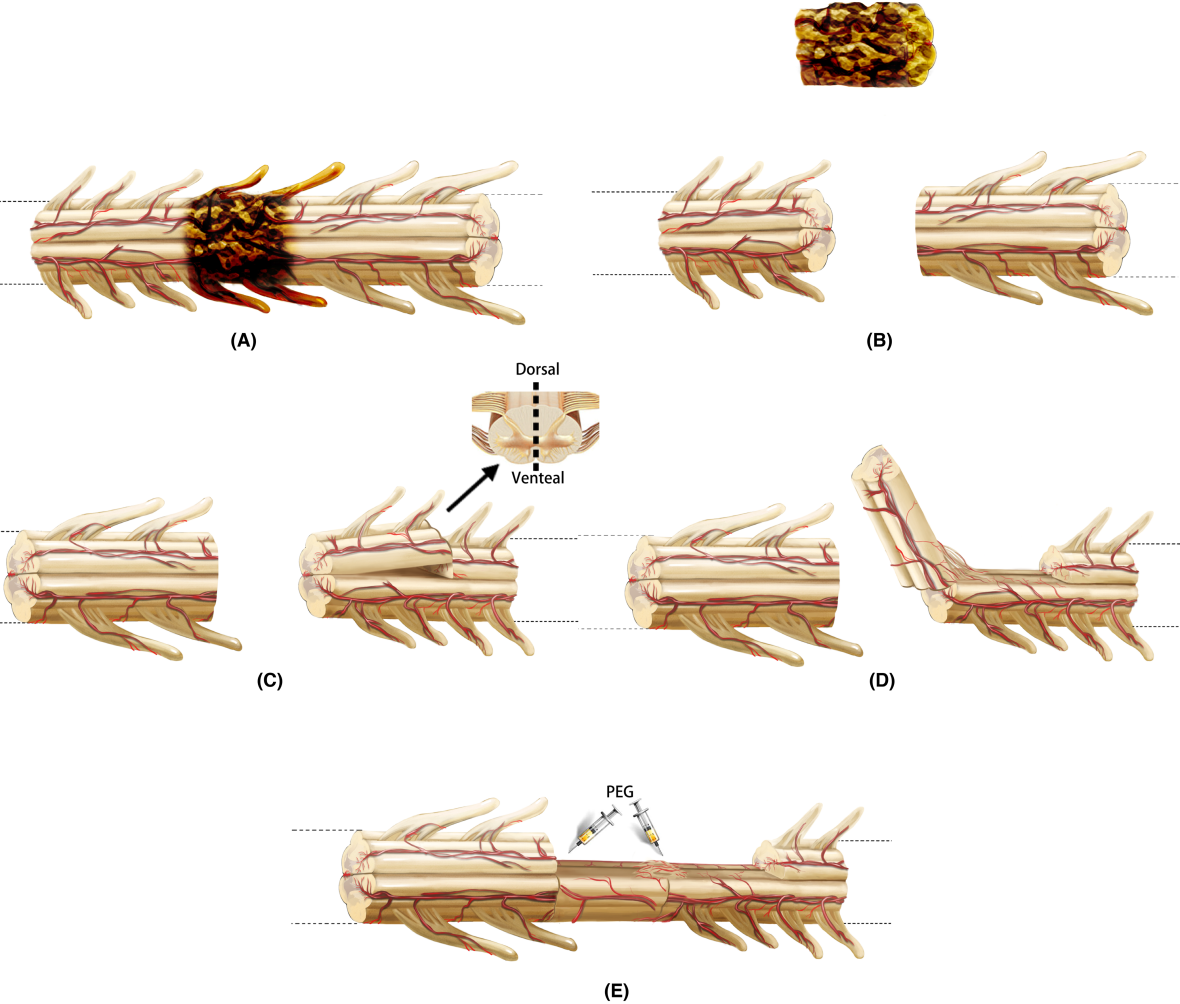
Key surgical steps of vSCT. First, the spinal cord injury area was removed to produce two fresh spinal cord stumps (A, B). Then, based on the effective length of the defect between two fresh spinal stumps, half of the spinal cord tissue with one side of the posterior spinal artery was cut from the distal or proximal spinal cord. The other side of the posterior spinal artery was maintained as the vascular pedicle to supply the blood flow (C). Then half of the spinal cord tissue was transplanted into the spinal cord defect area (D). PEG was topically applied to the two sites of spinal cord transection created after the transplantation to complete the SCF (E)

The participant was placed in a prone position under the general anesthesia. The skin and muscles overlying the thoracic spinal column were incised. A laminectomy was performed at the SCI level with a cutting ultrasonic scalpel (BoneScalpel^®^, Misonix) to expose the dura mater, which was then opened to expose the spinal cord. vSCT was performed, which is a new surgical treatment protocol that we originated and independently developed. The main surgical procedures of this operation are shown in Figure 1. An extremely sharp surgical knife (Shanghai Jingming Fine Technology Co.) was used to remove the SCI area of SCI to produce two acutely and very sharply transected spinal cord stumps. The removed spinal cord tissue was preserved for immunohistochemical staining. According to the effective defect length between the two spinal cord stumps, half of the spinal cord tissue with one side of the posterior spinal artery carefully preserved was cut from the distal or proximal spinal cord and transplanted to the gap to bridge the distal and proximal spinal cord stumps maintaining its arterial supply intact. The other side of the posterior spinal artery was maintained as the vascular pedicle to supply the blood flow to the transplanted spinal cord. Next, 10 ml of PEG‐600 (100%, Sigma‐Aldrich/Merck) was applied topically to the two sites of spinal cord transection created after the transplantation, and then the spinal pia mater was sutured under the microscope with a microsurgery suture thread with needle (7–0, COVIDIEN, USA) to stabilize the transplanted spinal cord. The dura mater was sutured with an artificial dural patch (Guanhao Biotech). A 3‐hole silicone drainage tube was placed into the muscular layer of the wound. The wound was closed in layers.

### 2.5 Safety evaluation

After the surgery, the participant's body temperature was measured three times a day. Blood routine examinations were performed every 3 days, and the surgical incision was observed daily to assess for postoperative infection. We also observed whether the participant had any symptoms of headache and dizziness, to evaluate whether there was a leakage of cerebrospinal fluid. Potential adverse reactions to PEG, such as urticaria, dermatitis, and even anaphylactic shock, were monitored daily.

### 2.6 Postoperative rehabilitation

After a professional assessment of spinal stability at 2 weeks postoperatively, all participants began a series of instrument‐assisted exercises, including the use of an exoskeleton robot (AiWalker, Beijing Ai‐Robotics Technology Co. Ltd) to assist walking (twice a day, 30 min each time, see online Video S1), an intelligent auxiliary mobile robot (BangBang, Shanghai Bangbang Robot Co. Ltd) or paraplegic standing frame to keep the participant standing (twice a day, 40 min each time), and a rehabilitation bicycle to exercise the lower extremity joints (twice a day, 1 h each time).

### 2.7 Immunohistochemistry

The removed spinal cord tissue was fixed with 4% paraformaldehyde for 24 h and then embedded in paraffin and horizontally cut into transverse slices of 7 μm thickness.

After removing the paraffin, the slices were quenched with endogenous peroxidase activity in 3% methanol hydrogen peroxide for 0.5 h. The antigen (https://www.abcam.com/protocols/ihc‐fixation‐protocol) was fixed in the citrate buffer (pH 6.0) in a microwave for 5 min. The slices were incubated with the primary antibodies (NF‐200, 1:50; MBP, 1:50, Abcam) overnight at 4°C, and then immersed with the biotinylated secondary antibodies (PV secondary antibody kit, Beijing Zhongshan Jinqiao Biotechnology Co. Ltd) for 0.5 h at room temperature. After incubating with peroxidase‐conjugated streptavidin, the immune complexes were visualized by incubating slices in the DAB system. The axons of the spinal cord were stained by the NF‐200‐specific antibody. The myelin sheaths were stained by an antibody specific for MBP.

### 2.8 Neurologic assessment

Neurologic evaluations by two evaluators blinded to the participants’ status were performed according to the International Standard of Neurological Classification for Spinal Cord Injury (ISNCSCI)/American Spinal Cord Injury Association (ASIA) classification of SCI.^22^ The ASIA impairment scale (Table 1), ISNCSCI motor score, and ISNCSCI pin prick and light touch scores were assessed.^22^

**TABLE 1:** ASIA impairment scale

ASIA impairment scale	
A	Complete. No sensory or motor function is preserved in the sacral segments S4–S5.
B	Incomplete. Sensory but not motor function is preserved below the neurologic level and includes the sacral segments S4–S5.
C	Incomplete. Motor function is preserved below the neurologic level, and more than half of the key muscles below the neurologic level have a muscle grade less than 3.
D	Incomplete. Motor function is preserved below the neurologic level, and at least half of key muscles below the neurologic level have a muscle grade greater than or equal to 3.
E	Normal. Sensory and motor function is normal.

Abbreviation: ASIA: American Spinal Injury Association.

### 2.9 Neurophysiological assessment

Somatosensory‐evoked potential and MEP were recorded before surgery and at 1, 3, and 6 months after surgery and analyzed with the NIM‐ECLIPSE^®^ System (Medtronic).

Cortical SSEPs were elicited by a 200 μs squarewave electrical pulse presented sequentially to the median and posterior tibial nerves. The stimulus frequencies were 3.96 Hz. Stimulus intensity was 20 and 30 mA to the median and posterior tibial nerves, respectively. Cortical potentials were recorded from monopolar needle electrodes placed at Cz’ for the posterior tibial nerve stimulation, C3’ or C4’ for the median nerve stimulation and referenced to Fpz (international 10–20 EEG system).

Motor evoked potentials were elicited with a brief duration of transcranial electrical pulses (pulse width =75 μs), high‐voltage (150 V) anodal electrical stimulus train. The stimulating electrodes were inserted over motor cortex regions at C3, C4 (international 10–20 EEG system), while the recording electrodes were positioned at the abductor pollicis brevis in the upper extremities and both tibialis anterior and abductor hallucis in the lower extremities.

### 2.10 Neuroimaging assessment

Participants were subjected to MRI and DTI using a 1.5 T MRI system (Ingenia 1.5, Philips) in the supine position. Sagittal, T2‐weighted, fast spin‐echo (TR =2500 ms; TE =110 ms; slice thickness =4 mm; slice gap =0.4; NSA =2), and axial single‐shot echo‐planar DTI (TR =7193 ms; TE =81 ms; voxel size =2.34 mm × 2.46 mm; slice thickness =5 mm; slice gap =0.06; NSA =4; diffusion direction number =15) sequences were acquired preoperatively and at 1, 3, and 6 months postoperatively in all participants. DTI original images were processed to produce color DTI maps.

### 2.11 Pain assessment

We elected to use the visual analog scale (VAS), because it is one of the most widely used methods to give valid and reliable assessments on experimental pain, as well as acute and chronic pain, in patients.^23^ The VAS consisted of a 10 cm horizontal line on a card with the words "no pain" and "worst pain ever" placed at the left‐ and right‐hand extremes of the line, respectively. The participants were instructed to mark the line at a point representing their pain intensity.^24^

### 2.12 Statistical analysis

All data were analyzed in SPSS Statistics software (SPSS 20.0, IBM). The Kolmogorov–Smirnov test and Shapiro–Wilk test for normality were used to assess the data distribution. All data exhibited a normal distribution (*p* > 0.05). Data are presented as the mean ± SD. Paired sample *t* tests were used for statistical analysis in the clinical trial. Statistical significance was set at a *p* < 0.05.

## 3 RESULTS

### 3.1 Participant enrollment

**FIGURE 2:**
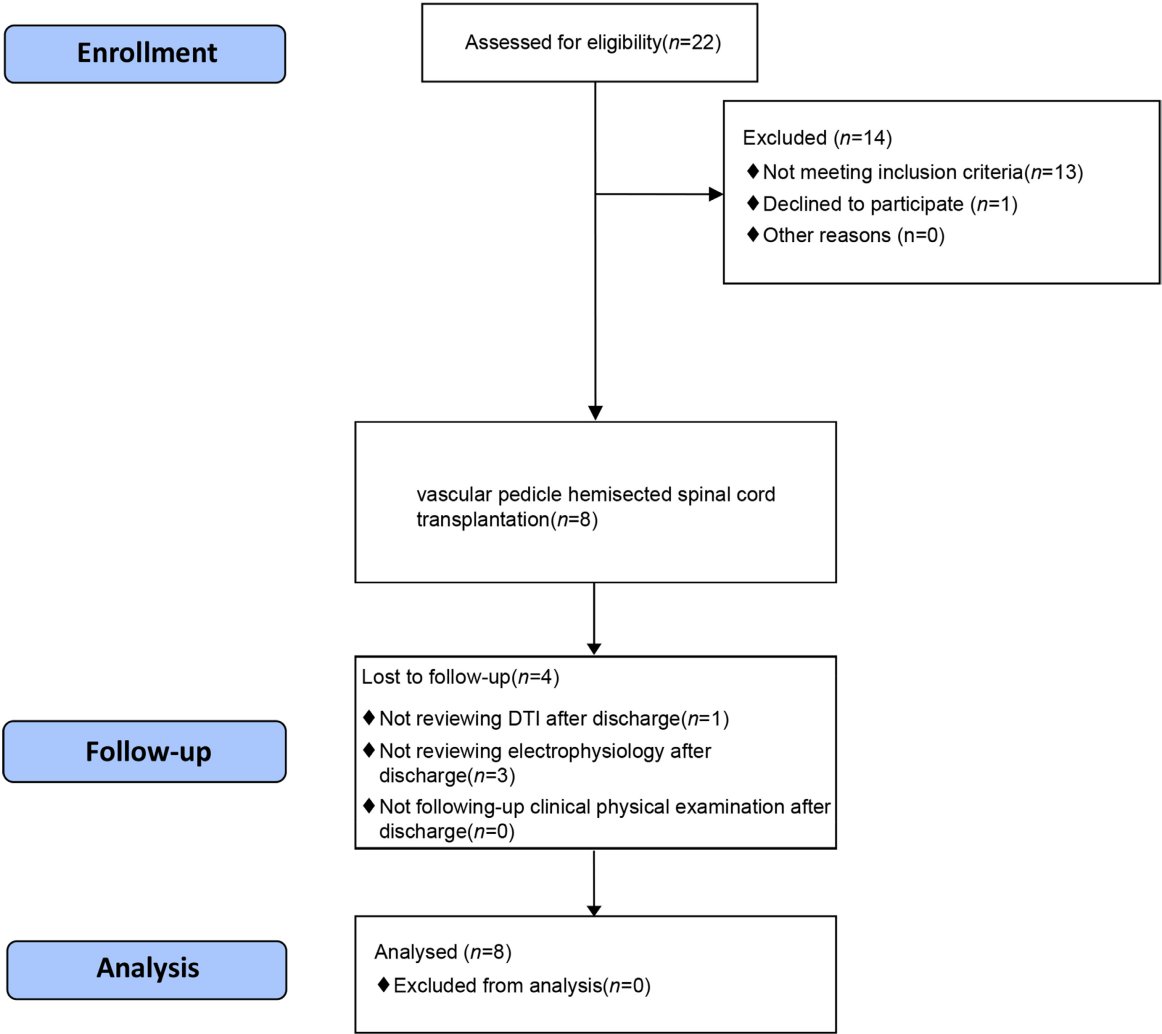
CONSORT flow diagram

We enrolled eight participants (six men and two women) into the clinical trial (Table 1, Figure 2). All participants were classified as ASIA Impairment Scale grade A and had complete injuries in the thoracic spinal cord segment.

The entire operation of vSCT took approximately 4 h. The mean length of spinal cord removed was 5.5 cm (Table 2, maximum 7 cm, minimum 1.5 cm). After vSCT, all participants tolerated the perioperative period safely without complications, such as high fever, wound infection, and cerebrospinal fluid leakage. In addition, none of the participants had clinically important adverse reactions to PEG over the 6 months after the surgery.

**TABLE 2:** Clinical features of the participants

Participant ID	Sex	Age	Months post‐SCI	SNL	Length of SCI tissue (cm)
SCF001	Female	26	16	T6	6
SCF002	Male	14	15	T4	6
SCF003	Male	37	55	T9	6.5
SCF004	Male	46	18	T10	6
SCF005	Male	33	18	T10	1.5
SCF006	Male	44	65	T8	5
SCF007	Male	40	30	T4	7
SCF008	Female	34	30	T6	6
Mean ± SD	‐	34.25 ± 10.38	32 ± 18.35	‐	5.5 ± 1.7

Abbreviations: SCF, spinal cord fusion; SCI, spinal cord injury; SNL, single neurologic level.

### 3.2 Immunohistochemistry

**FIGURE 3:**
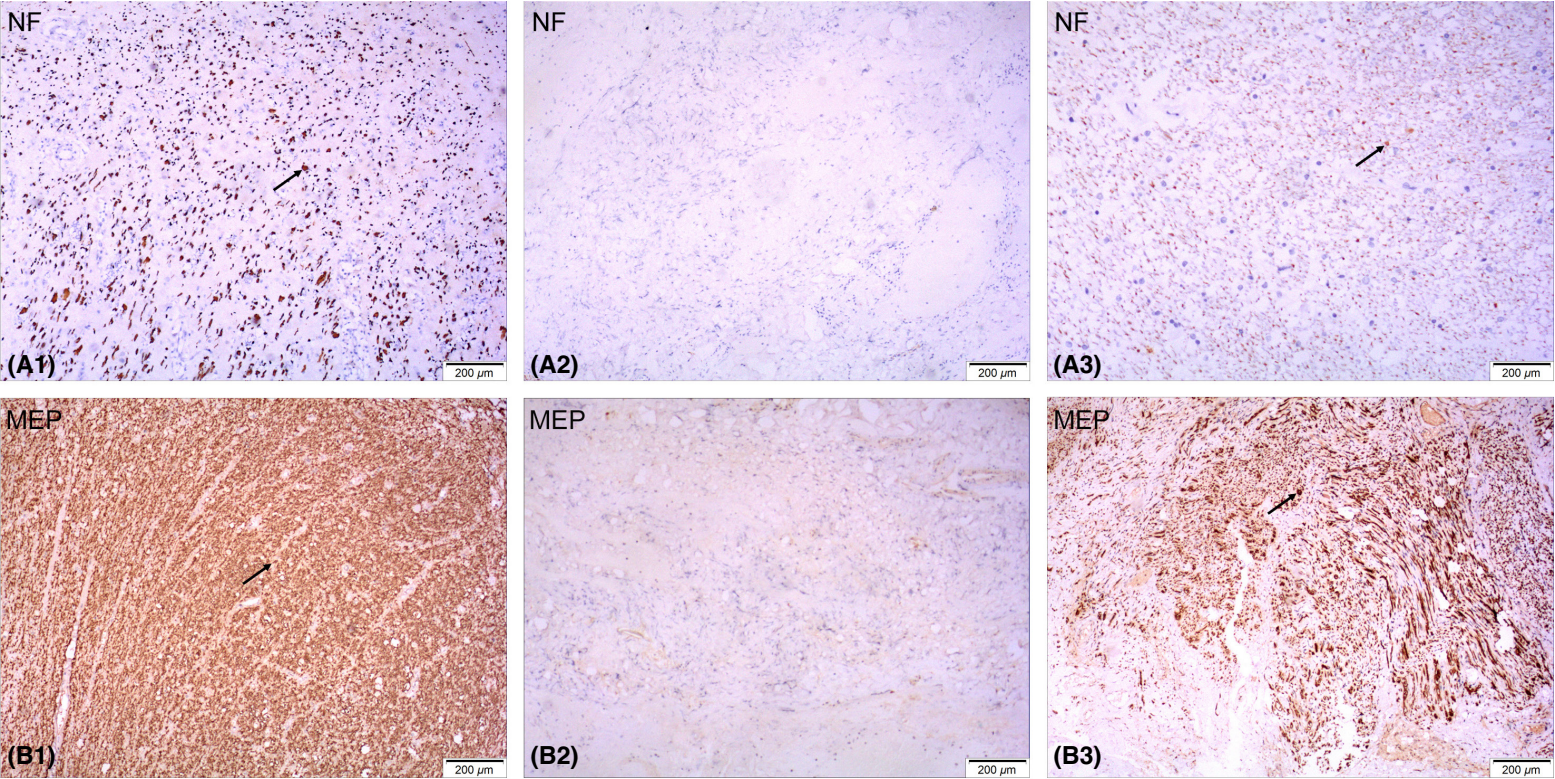
Immunohistochemical staining of spinal cord tissue removed during the surgery. NF‐200‐positive axons and MBP‐positive myelin sheaths can be observed at the distal and proximal ends of the tissue (arrows in A1, B1, A3, B3) but not in the center of the tissue (A2, B2)

The spinal cord tissue removed during the surgery was stained with immunohistochemical staining and imaged at 40× under an Olympus microscope (Olympus IX73). Immunohistochemistry showed that NF‐200‐positive axons and MBP‐positive myelin sheaths could be observed at the distal and proximal ends of the tissue (Figure 3A1,A3,B1,B3), but not in the center of the damaged tissue (Figure 3A2,B2).

### 3.3 Neurophysiological assessment

Preoperatively, the SSEPs of bilateral median nerve were normal in these participants with paraplegia, and the SSEPs of bilateral posterior tibial nerve were completely absent. In addition, the MEPs recorded at the abductor pollicis brevis muscles in the bilateral upper extremities were normal, and the MEPs recorded at both tibialis anterior and abductor hallucis muscles in the bilateral lower extremities were completely absent.

**FIGURE 4:**
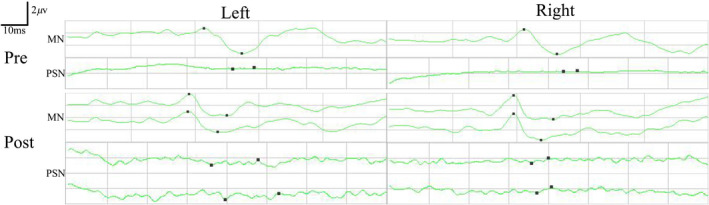
Pre‐ and postoperative SSEPs of median nerve (MN) and posterior tibial nerve (PSN) in participant SCF006. Postoperative SSEPs of both lower extremities PSN did not show significant recovery compared with preoperative SSEPs. Pre, preoperatively; Post, postoperatively

**FIGURE 5:**
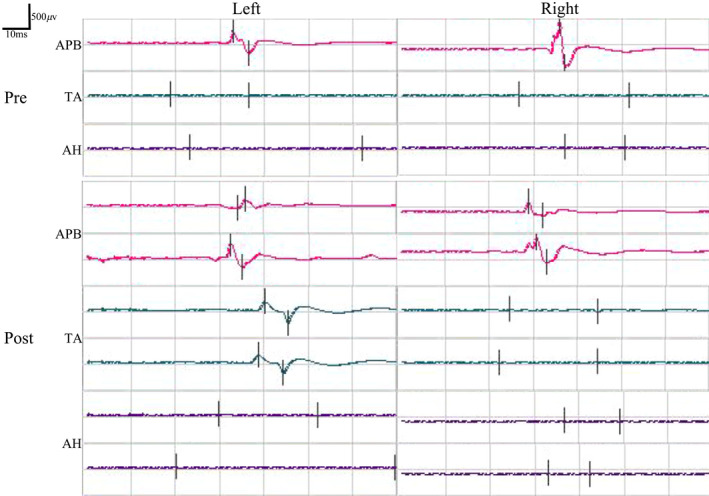
Pre‐ and postoperative MEPs recorded at the abductor pollicis brevis (APB), tibialis anterior (TA), and abductor hallucis (AH) by the transcranial electrical stimulation in participant SCF006. MEPs recorded at the left tibialis anterior showed sign of recovery postoperatively. Pre, preoperatively; Post, postoperatively

The preoperative SSEPs and MEPs of participant SCF006 were similar to those of the other seven participants (Figures 4 and 5). Although postoperative SSEPs of bilateral posterior tibial nerve did not show significant recovery compared with preoperative SSEPs in participant SCF006, the restoration of MEPs recorded at left tibialis anterior was noted at 1 month after the surgery (the right MEPs did not recover) (Figures 4 and 5).

### 3.4 Neuroimaging assessment

**FIGURE 6:**
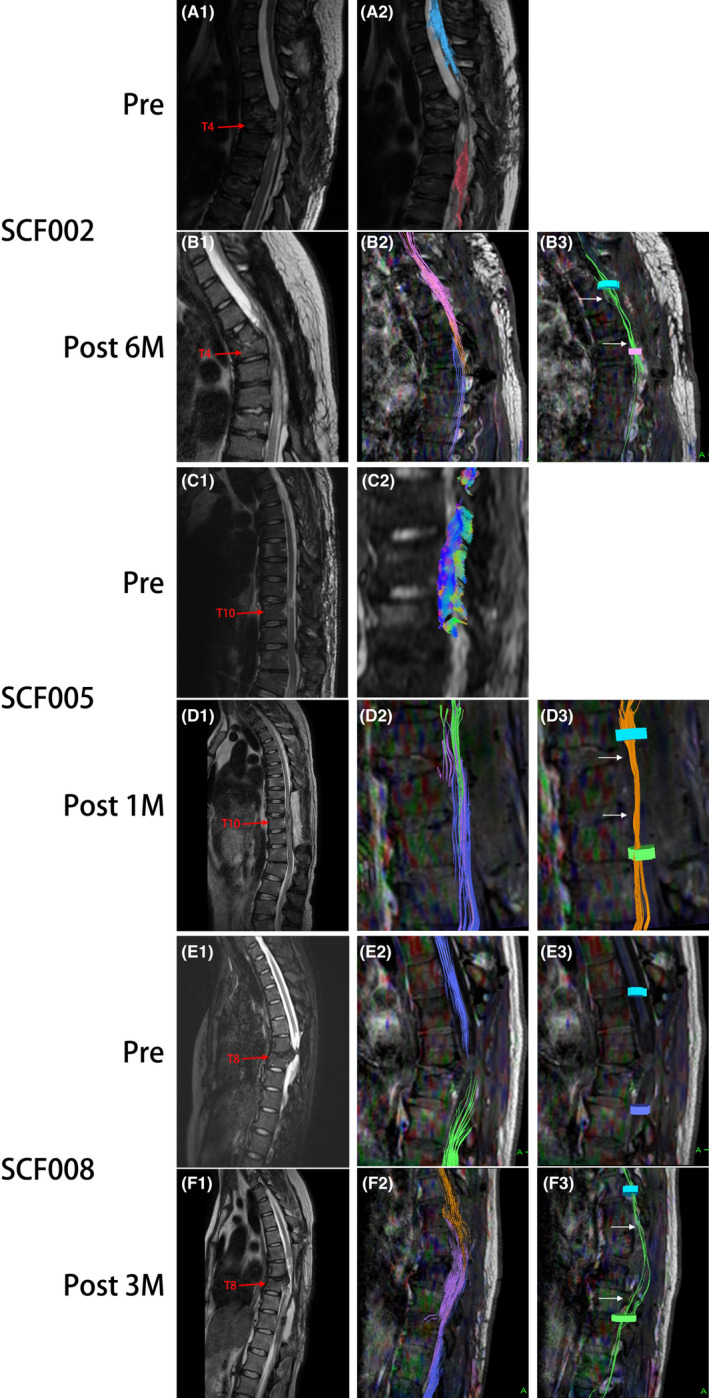
Representative neuroimaging images of participants SCF002, SCF005, and SCF008 treated with vSCT. Preoperative T2‐weighted MRIs images show significant spinal cord injury (A1, C1, E1). DTI images show complete disruption of the spinal cord fibers (A2, C2, E2, E3). Postoperative T2‐weighted MRI images show the vascularized transplanted spinal cord in the original SCI area (B1, D1, F1). The three colored nerve fibers shown by DTI images represent nerve tracing and imaging of the distal and proximal spinal cord and the vascularized transplanted spinal cord (B2, D3, F3). DTI images show the reconnection of some nerve fibers to restore the nerve continuity of the spinal cord (B3, D3, F3). Pre, Preoperatively; Post 1 M, 1 month postoperatively; Post 2 M, 2 months postoperatively; 6 M: 6 months postoperatively

Preoperatively, T2‐weighted MRI scans from all participants showed markedly abnormal signal intensity in the SCI area (Figure 6A1,C1,E1). In addition, DTI showed no continuity of nerve fibers in the proximal and distal spinal cords of these participants (Figure 6A2,C2,E2,E3).

MRI and DTI were repeated on all participants after the surgery. T2‐weighted MRI scans of all participants showed that the vascularized transplanted spinal cord could be observed at the original SCI site (Figure 6B1,D1,F1). In addition, the DTI of three participants also showed important positive results, including the fibers tracked at the operating site and the overlapped three bundles of nerve fibers above and below the operating site (Figure 6B2,D2,F2). The fibers were tracked at two selected planes above and below the operating site to show the fibers that crossed two transectional sites (arrows in Figure 6B3,D3,F3). The DTI of participants SCF002, SCF005, and SCF008 showed reconnection of some nerve fibers, restoring some neural continuity of the spinal cord (Figure 6B3,D3,F3).

### 3.5 Neurologic assessment

Six participants resumed sweating below the single neurologic level. Three participants had improved bladder and/or bowel functions. The participants could feel the fullness of their bladder and/or bowel which they were unable to do preoperatively, and stool character was improved from dry and hard to wet and soft, which they were unable to do preoperatively. One participant re‐experienced the dysmenorrhea symptoms during menstruation she had lost after the SCI (Table 3). More importantly, participant SCF008 was able to autonomously flex and extend the ankle and toe joints of both lower extremities at 1 month postoperatively and maintained this degree of motor function 6 months after the surgery (online Video S1). The ISCNSCI lower extremity motor scores in participant SCF008 were restored from 0 to 16 (normal is 50). The ASIA grade of the participant was improved from A to C (Table 4).

**TABLE 3:** Recovery condition of autonomic nerve function in paraplegic participants at 6 months postoperatively

Participant ID	Recovery condition of autonomic nerve function
SCF001	Sweating function below the single neurologic level restored; dysmenorrhea appeared; bowel function improved
SCF002	Sweating function below the single neurologic level restored; bladder and bowel function improved
SCF003	Sweating function below the single neurologic level restored
SCF004	Bladder function improved
SCF005	Sweating function below the single neurologic level restored
SCF006	Sweating function below the single neurologic level restored
SCF007	‐
SCF008	Sweating function below the single neurologic level restored

Abbreviation: SCF, spinal cord fusion.

**TABLE 4:** Sensory and motor function assessment according to the International Standards for Neurological Classification of Spinal Cord Injury

Participant ID	Before surgery						6 months after surgery					
	SNL	ISCNSCI UEMS	ISCNSCI LEMS	ISCNSCI light touch scores	ISCNSCI pinprick scores	ASIA grade	SNL	ISCNSCI UEMS	ISCNSCI LEMS	ISCNSCI light touch scores	ISCNSCI pinprick scores	ASIA grade
SCF001	T6	50	0	52	52	A	T6	50	0	52	52	A
SCF002	T4	50	0	48	48	A	T4	50	0	48	48	A
SCF003	T9	50	0	64	64	A	T9	50	0	64	64	A
SCF004	T10	50	0	70	70	A	T5	50	0	50	52	A
SCF005	T10	50	0	70	70	A	T8	50	0	64	64	A
SCF006	T8	50	0	60	60	A	T4	50	0	44	44	A
SCF007	T4	50	0	44	44	A	T4	50	0	44	44	A
SCF008	T6	50	0	52	52	A	T6	50	16	52	52	C

Abbreviations: ASIA, American Spinal Injury Association; ISCNSCI, International Standard of Neurological Classification for Spinal Cord Injury; LEMS, motor score of the lower extremities; SCF, spinal cord fusion; SNL, single neurologic level; UEMS, motor score of the upper extremities.

### 3.6 Pain assessment

**FIGURE 7:**
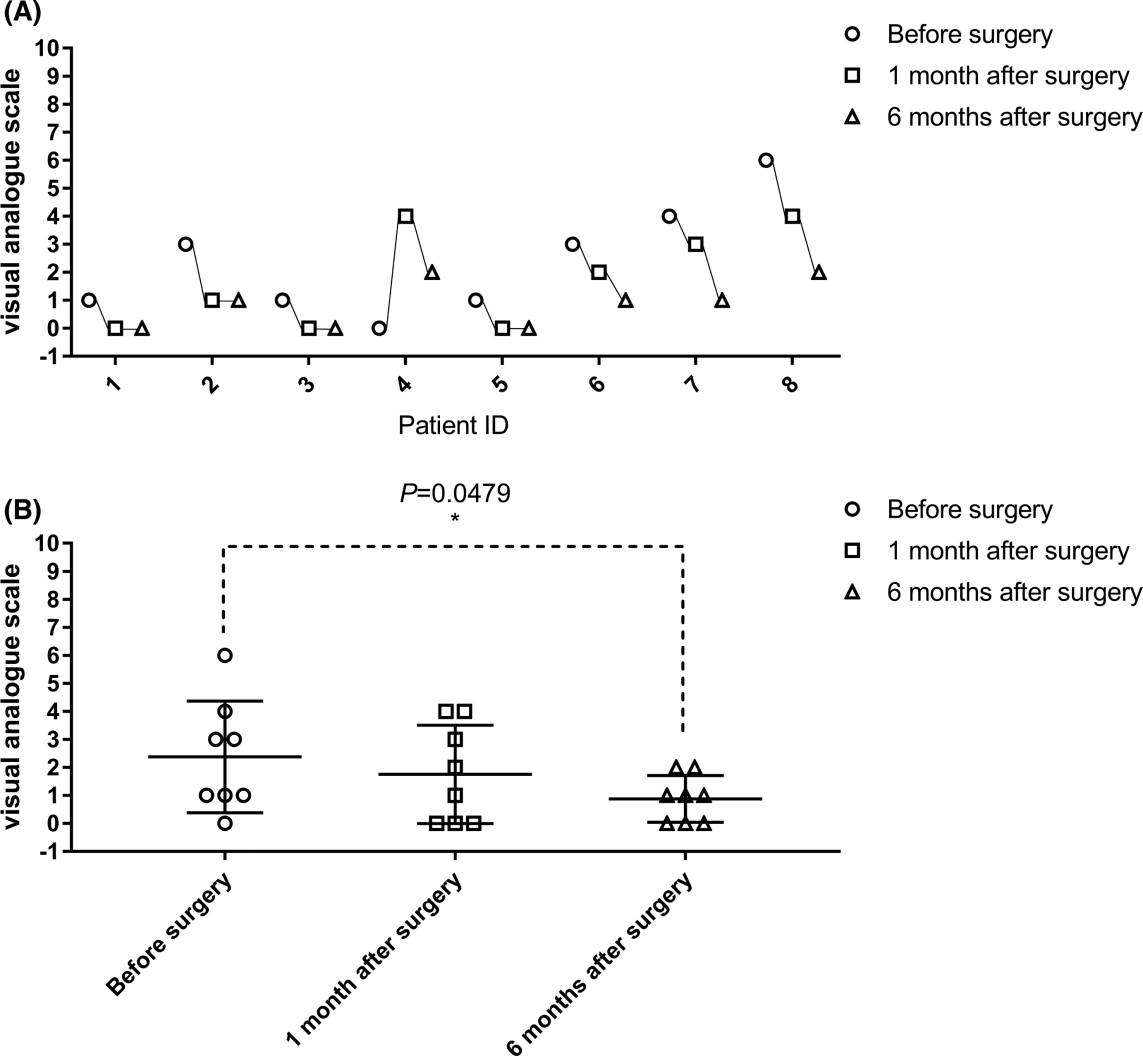
Evaluation of cord central pain in participants with paraplegia before, 1 month, and 6 months after the surgery (visual analog scale, VAS). The VAS in participant SCF004 at 1 month after the surgery was increased compared with that before the surgery, while the VAS decreased at 6 months after the surgery. The VAS of four participants (SCF002, SCF006, SCF007, and SCF008) after the surgery were gradually decreased compared with that before the surgery. The remaining participants were unchanged (1–8 on the horizontal axis represents SCF001‐SCF008, see in A). The difference between VAS before and 6 months after the surgery was statistically significant (*p* < 0.05) (B)

Cord central pain is one of the main and most intractable complications of patients with paraplegia after a SCI and seriously affects their quality of life.^25^ In this clinical trial, all eight paraplegic participants had some degree of spinal cord pain preoperatively. Seven paraplegic participants with cord central pain had improved symptoms after the surgery. The mean VAS of all participants at 1 and 6 months after the surgery gradually decreased compared with that before the surgery (1.75 at 1 month after the surgery and 0.86 at 6 months after the surgery vs. 2.38 before the surgery, Table 5, Figure 7). The difference between the VAS score before and 6 months after the surgery was statistically significant (*p* < 0.05; Figure 7).

**TABLE 5:** Visual analog scale for cord central pain in paraplegic participants

Participant ID	VAS		
	Before surgery	1 month after surgery	6 months after surgery
SCF001	1	0	0
SCF002	3	1	1
SCF003	1	0	0
SCF004	0	4	2
SCF005	1	0	0
SCF006	3	2	1
SCF007	4	3	1
SCF008	6	4	2
Mean ± SD	2.38 ± 2.00	1.75 ± 1.75	0.86 ± 0.83

Abbreviations: SCF, spinal cord fusion; SCF, spinal cord fusion; VAS, visual analog scale.

## 4 DISCUSSION

It is well known that glial scar formation is an important part of the pathophysiological mechanism of SCI. In chronic SCI, glial scarring creates a physical and chemical barrier to axon regeneration in the injured area.^26^ Therefore, scar removal is one of the necessary steps during the clinical treatment of SCI. However, removal of scar tissue results in a large gap between the two severed cord stumps. In the present vSCT clinical trial, the average length of SCI area (glial scar) was 5.5 cm. Therefore, it is unrealistic to expect nerve regeneration to restore nerve continuity across the gap only by surgical resection of the scar. In the current study, our team proposed a new surgical procedure called vSCT. We removed the SCI area (glial scar) to produce two acutely transected spinal cord stumps. Then, half of the spinal cord tissue with the posterior spinal artery was cut from the distal or proximal spinal cord stumps and transplanted to bridge the distal and proximal stumps. Finally, PEG fusion was topically applied at the axon stumps at the sites of the two spinal cord transections to restore spinal nerve continuity according to the GEMINI SCF protocol.

The difference between the clinical paraplegic humans and the beagles is that the beagle model was constructed with an acute SCI, while the participants in the clinical trial had chronic SCI. The vSCT operation in humans requires surgical of the spinal glial scar as determined by MRI/DTI results combined with the observation of the blood supply in the distal and proximal spinal cord stumps. In our patients, immunohistochemical staining of the removed glial scar tissue showed that nerve fibers and myelin tissue structures could be observed at the two edges but not the center of the glial scar tissue. Confirming that the proximal and distal spinal cord stumps contained many viable nerve fibers. In addition, during the removal of glial scar tissue, we fully understood that small parts of normal spinal cord tissue and nerve roots near the scar might need to be removed. It is this reason that some participants had a slight ascent in the single neurologic levels after the surgery. Studies of dorsal rhizotomies in patients with chronic pain have verified that nearby roots could compensate for such resulting deficits.^27^

According to previous experimental work, several of the keys to successful SCF are an extremely sharp, non‐traumatic transection of the spinal cord that results in minimal local damage to the gray and white matter and then ensuring no gap between approximated spinal cord tissue.^3^, ^4^, ^5^, ^6^ The typical force generated by this type of sharp transection is <10 N.^28^ The length of the vascularized transplanted spinal cord segment in our patients was slightly longer than the defect created by the scar removal. This ensured that the vascularized transplanted spinal cord was well approximated with the distal and proximal spinal cord stumps with no gap.

The GEMINI SCF protocol also mentions two sets of fiber tracts from the brain to the spinal cord to regulate the voluntary function of the extremities. The first system, the pyramidal tract, consists of long, fast signal‐transmitting neurons that connect the nerve cells in the brain cortex to the spinal cord nerve cells that allow rapid transmission of volitional signals.^5^ The PEG treatment allows acute fusion of an unknown number of transected white matter fibers, including the pyramidal tract,^13^, ^14^, ^15^ as shown in the postoperative DTI results of participants in this clinical trial. Among the paraplegic participants who participated in this clinical trial, we found that in three participants the postoperative DTI (Figure 6, participant SCF002, SCF005, and SCF008) already showed the restoration of spinal cord neural continuity and the PEG‐fused transected white matter fibers.

The second system is a gray matter‐based network of propriospinal neurons, called the CTRPS pathway.^3^, ^4^, ^6^ In addition to the first system, this intraspinal network of propriospinal neurons plays a critical role in the motor reflexes, voluntary movement, and sensory processing, as well as in the functional recovery after SCI.^16^ Even if no axons of the pyramidal tract could be fused during the GEMINI protocol by PEG, the entire recovery could hinge on PEG fusion of axon stumps of propriospinal neurons. In addition, these same propriospinal neurons, along with others in proximity that were not damaged by the extra‐sharp blade, could regrow (sprout) their fibers immediately and re‐establish contacts between the apposed interfaces.^5^, ^29^ In the central nervous system, DTI can only trace and image white matter fibers.^19^, ^20^, ^21^ We have not yet been able to determine whether DTI can image the continuity of the CTRPS propriospinal neurons network. In our beagle experiments, the DTI of one beagle (PEG group) did not show recovery of neural continuity of the spinal cord, but after the vSCT, this beagle regained motor function of the hind limbs and the cBBB score was restored from 0 to 11 (unpublished data). DTI and behavior studies of the beagle demonstrated that these propriospinal neurons in the gray matter of the spinal cord stumps after this sharp transection regrew (sprouted) immediately and established neural continuity between the apposed interfaces after surgery. At this point, PEG may have led to the fusion of the axon stumps of the propriospinal neurons to re‐establish neural continuity. Therefore, we cannot exclude the possibility that participants might restore cord neural continuity through the CTRPS pathway in the clinical trial.

In this clinical trial, postoperative DTI and electrophysiological results have indicated restoration of spinal cord continuity and neural function in some participants. Restoration of spinal cord continuity is a prerequisite for the recovery of lost sensory and motor functions below the single neurologic level. However, the paraplegic participants in the clinical trial all had chronic spinal cord injuries, with an average time after injury nearly 3 years. These participants had various degrees of spinal cord and muscle atrophy, which might make it impossible for participants in clinical trials to restore sensory and motor functions either as quickly as beagles (see Appendix [Link], [Link], [Link], [Link]). In addition to the SCF clinical trial, the encouraging results of other case studies of the treatment using similar though not PEG‐induced treatments for chronic SCI treatment also support this trial. In 2005, a patient with a spinal cord defect of 4 cm was treated with an omental–collagen bridge procedure at 42 months after SCI; the patient was able to walk with a walker and soft knee braces 4 years postoperatively.^30^ In 2014, a patient with a spinal cord defect of 10 mm was treated by transplantation of bulbar olfactory ensheathing cells with peripheral nerve bridging at 21 months after SCI; the patient was able to perform lower extremity adduction, hip flexion, and knee extension about 1 year postoperatively.^31^ In 2016, five patients with an average spinal cord defect of 2.9 cm at an average of 13 months post‐SCI were treated by the NeuroRegen scaffold bridge along with autologous bone marrow mononuclear cells; certain patients had some recovery of autonomic nerve function at the 1‐year postoperative observation.^32^ In our trial, eight chronic SCI participants were treated with vSCT at an average of 32 months after SCI with an average spinal cord defect of 5.5 cm; the follow‐up observation period of only 6 months, however, is not sufficient, and long‐term observation are necessary and will occur.

Another possibility is that, the fusion of sensory and motor nerve fibers achieved by vSCT may not lead to fusion with the correct nerve fibers across the site of transection, leading to a mismatch of appropriate fibers. We know that the nervous system is quite plastic with recovery from any anatomic disruption of the spinal cord requiring a “rewiring”^33^; this reorganization of the central nervous system may explain the long‐time window of recovery of sensory and motor function in the clinical trial participants. Whether any of our patients will benefit in terms of motor and sensory function from a longer postoperative recovery period we do not know, though we are optimistic.

In addition to complete loss of motor/sensory function below the single neurologic level, many patients, perhaps most with SCI have some degree of autonomic dysfunction. In this clinical trial, three of the participants treated with vSCT had improved bladder and/or bowel function. In some, the skin below the single neurologic level recovered sweating function. Equally important, our clinical trial showed that vSCT could relieve many of the symptoms of cord central pain in our paraplegic participants, a benefit well‐received. Seven of these eight participants with cord central pain symptoms had their symptoms of cord central pain were relieved to some extent; however, one participant with no preoperative symptoms of cord central pain symptom developed the cord central pain symptom (VAS: 4) after the surgery. This might be because we fashioned the transplanted cord bridge from the proximal spinal cord site (the preoperative MRI showed substantial atrophy of the distal cord). This may have caused damage to the proximal spinal cord tissue which resulted in postoperative cord central pain. Luckily, after the rehabilitation treatment, the cord central pain symptoms partly were relieved at 6 months postoperatively with the VAS score reducing to two.^34^

Based on the results of this clinical trial, we plan to try to optimize the experimental protocol before expansion of the clinical trial. We will optimize the fusogen (PEG) and apply electrical stimulation to improve surgical outcomes.^35^, ^36^ In addition, mental imagery (MI) therapy will be added to our postoperative rehabilitation program. MI refers to the active process by which humans relive the sensations with or without external stimuli^37^; moreover, MI as a rehabilitation method can improve cord central pain in SCI.^38^ Also, we will review the operative technique to reduce surgical injury, thereby minimizing postoperative scarring. Finally, we propose allograft spinal cord transplantation as another potential treatment for SCI.^39^ Theoretically, the number of nerve fibers and propriospinal neurons in an allograft of spinal cord is about twice that of vSCT. Therefore, there will be more nerve fibers to potentially reconnect, thereby leading to a more optimal, faster, and better postoperative recovery of neurologic function.

## 5 CONCLUSION

In summary, we demonstrated the clinical feasibility and safety of vSCT through a prior preclinical trial in beagles and a clinical trial in eight paraplegic participants. PEG treatment appears to be able to fuse the axon stumps and restore the continuity of some nerve fibers during vSCT. vSCT could provide the basis of anatomy, morphology, and histology to potentially restore the motor and sensory functions of appropriately selected patients with paraplegia. vSCT also appears able to ameliorate much of the cord central pain and improve the autonomic nervous function. In addition, we have been evaluating allograft spinal cord transplantation as another option during the SCF to treat paraplegic patients.^39^

## CONFLICTS OF INTEREST

The authors declare that they have no conflict of interest.

## Supporting information

App S1

Fig S1

Fig S2

Fig S3

Video S1

## Data Availability

The data that support the findings of this study are available from the corresponding author upon reasonable request.
