# Supplementary material for: Partial restoration of spinal cord neural continuity via vascular pedicle hemisected spinal cord transplantation using spinal cord fusion technique
Source: CNS Neurosci Ther. 2022 May 12;28(8):1205–17. doi: 10.1111/cns.13853 (PMC9253790; doi:10.1111/cns.13853)
Supplement: Supplementary file 1 — App S1 [file CNS-28-1205-s001.docx]

Supplementary material to:

Partial Restoration of Spinal Cord Neural Continuity via Vascular Pedicle Hemisected Spinal Cord Transplantation Using Spinal Cord Fusion Technique

**METHODS**

**1. Animals**

All animal procedures were reviewed and approved by the Institutional Animal Care and Use Committee of Harbin Medical University (IACUC) and the Institute of Laboratory Animal Science of China (A5655-01) and were in accordance with Directive 2010/63/EU of the European Parliament. Eight-month-old female beagles (8 kg) were used in this study due to their docility and greater resistance to urinary tract infections. Animals (Qingdao Agricultural University, Qingdao, Shandong, China) were housed comfortably with a light/dark cycle of 12 h/12 h and fed *ad libitum*. Beagles were randomly assigned to two groups: PEG group (n=4) and control group (n=4).

**2. Surgery**

The paraplegic animal model was followed as previously described^1-3^. Briefly, after anesthesia, the beagles were placed in a prone position on the operating table. The skin and muscles overlying the thoracic spinal column were incised. Laminectomy was performed at T10. Under a surgical microscope, the dura mater was opened, and an extremely sharp surgical knife (Shanghai Jingming Fine Technology Co., Shanghai, PRC) was used to remove about 1 cm of the spinal segment, resulting in two spinal cord stumps, which is the model of acute spinal cord injury of the beagles. According to the effective distance between the distal and proximal stumps of the spinal cord, the distal 1.5 cm of the caudal spinal cord was hemisectioned longitudinally. A 1.5 cm spinal cord segment with one side of the posterior spinal artery was transplanted to the gap to bridge the distal and proximal spinal cords. The other side of the posterior spinal artery was maintained as the vascular pedicle to supply the blood flow to the transplanted spinal cord. Therefore, two sites of spinal cord transection were produced after transplantation. Immediately thereafter, the beagles received either 0.9% NaCl (2 mL) or PEG-600 (2 mL) applied and left topically on two sites of spinal cord transection via a syringe. Standard closure by layers was followed.

**3. Motor assessment**

Two trained and blinded examiners used the 20-point (0–19) canine Basso-Beattie-Bresnahan rating scale to assess the beagle's hindlimb movement, coordination, and trunk posture, as well as stability in an open field^4^. The scoring time was arranged at 1, 2, 3, 4, 6, 8, 12, 16, 20, and 24 weeks after the surgery.

**4. Neuroimaging assessment**

All animals were subjected to diffusion tensor imaging (DTI) using a 3.0 T magnetic resonance imaging (MRI system) (Achieva 3.0, Philips, Amsterdam, The Netherlands) in the prone position. Axial single-shot echo-planar DTI (TR = 6100 ms; TE = 93 ms; voxel size = 2 mm x 2 mm; slice thickness = 2 mm; slice gap = 0; NSA = 2; diffusion direction number = 15) sequences were acquired twice at two weeks postoperatively in all animals.

**5. Tissue preparation**

At 24 weeks after the surgery, all beagles were killed with an overdose of pentobarbital sodium and transcardially perfused with saline followed by 4% paraformaldehyde. The vascularized transplanted spinal cord tissue from the T10 level was removed. The tissue was fixed with 4% paraformaldehyde for 24 h, embedded in paraffin, and horizontally cut into transverse slices of seven μm thickness.

**6.** **Immunohistochemistry**

Immunohistochemical methods for neurofilament and myelin basic protein (MBP) were identical to those described in clinical trials (see MANUSCRIPT). The spinal cord slices were imaged at 400x under an Olympus microscope (Olympus IX73, Tokyo, Japan). The axons of the spinal cord were stained by the NF-200 specific antibody. The myelin sheaths were stained by an antibody specific for MBP. Image-Pro Plus 6.0 software was used to calculate and analyze the NF-200 and MBP positive staining density.

**7. Statistical Analysis**

All data were analyzed in SPSS Statistics software (SPSS.20, IBM, New York, USA). The Kolmogorov-Smirnov test and Shapiro-Wilk test for normality were used to assess the data distribution. The data of cBBB scores did not exhibited a normal distribution (*P* < 0.05). The data are presented as the median ± quartile, and the Mann–Whitney U-test was used for statistical analysis. The data of the positive staining density exhibited a normal distribution (*P* > 0.05). The data are presented as the mean ± SD, and unpaired sample *t* test was used for statistical analysis. Statistical significance was set at a *P* < 0.05.

**RESULTS**

**1. Behavioral assessment of beagles**

All the beagles in the study, four PEG-group beagles and four control beagles, survived the surgery. At 24 weeks post-surgery, sphincter control of the bladder was not sufficiently restored in either beagle group, and continued assistance was required to empty the bladder, as seen in previous research^2,5^. Massage twice a day was required for defecation.

Before the surgery, all beagles had normal cBBB scores (score: 19). All beagles underwent the surgery and as expected, showed complete paraplegia immediately after the surgery (score: 0). No significant recovery of both hind limbs was observed in any of the control group beagles during the entire observation period (24 weeks). In contrast, two to three weeks after the surgery, PEG group beagles showed slight or even extensive movements in the hip, knee, and ankle joints of both hind limbs. Over time, beagles in the PEG group continued to have steady recoveries in bilateral hind limbs (Fig. S1). At 24 weeks post-surgery, two beagles scored 12 points, one scored 11 points, and one scored 10 points. All four beagles in the PEG group were able to support their weight to stand. Among them, three beagles could ambulate frequently, and one beagle could ambulate occasionally (Table S1).

The cBBB scores of the two groups showed a statistically significance difference starting four weeks after the surgery (Mann–Whitney U-test: *P* < 0.05, Table S1, Fig. S1). In addition, 24 weeks post-surgery, beagles in the PEG treatment group had a median score of 11.5 (range: 10 – 12) versus 4 (range: 3 – 5) in the control group (Mann–Whitney U-test: *P* < 0.05, Table S1, Fig. S1).

**2. Neuroimaging assessment of beagles**

MRI and DTI were used to show the nerve fibers *in vivo*. DTI is the most extensively used technique worldwide to study the microstructural properties of white matter of the central nervous system (CNS)^6-8^. In the PEG group, the nerve fiber tracts of proximal and distal spinal cord were connected across the operative area, restoring neural continuity of the spinal cord (Fig. S2A, B). In contrast, the DTI of the control group beagles showed that the nerve fibers of the proximal and distal spinal cord never connected and there were gaps in the operative area (Fig. S2C, D).

**3.** **Immunohistochemistry**

Immunohistochemistry was performed to analyze the survival of nerve fibers in the vascularized transplanted spinal cord. The NF-200-positive axons and MBP-positive myelin sheaths were observed in the vascularized transplanted spinal cord of the PEG group (Fig. S3A, C), but were rarely observed in the control group (Fig. S3B, D). The positive staining densities of NF-200 and MBP in the PEG group was significantly higher than those in the control group (p<0.05, Fig. S3E, F).

**DISCUSSION**

Polyethylene glycol (PEG) is a relatively inexpensive, stable, non-toxic, and completely biocompatible, water-soluble linear polymer. It is formed by the ring-opening polymerization of anions of ethylene oxide, and its molecular weight ranges from 0.4 to 100 kDa^9^. PEG is approved by the Food and Drug Administration (FDA) in the United States as a preservative additive prior to organ transplantation to limit cold ischemia/reperfusion injury^10^.

Prior to the clinical trial of vascular pedicle hemisected spinal cord transplantation (vSCT), a related preclinical study was carried out using beagles as the experimental model. The results suggested that the permanent paralysis after removing about 1 cm of spinal tissue could be prevented in part by immediate, topical, and intraoperative application of a fusogen (PEG) in the regions of the spinal cord transection. The cBBB scores of the animals treated with PEG were significantly higher than those treated with normal saline 24 weeks after operation. The 24 weeks postoperative cBBB scores of the PEG group in this current study were very similar to findings from our previous canine study of a completely transected spinal cord treated with PEG (median scores: 11.5 versus 11)^2^. Immunohistochemical results and DTI of the animals also supported the recovery of motor function in the PEG group (Fig. 3-4). In addition, none of the beagles died after the vSCT surgery, nor developed central nerve pain symptoms or any PEG-related adverse reactions. The success of vSCT in beagles laid a good foundation for the clinical transformation of this procedure.

**REFERENCES**

1. Ren S, Liu ZH, Wu Q, et al. CNS Neurosci Ther. 2017;23(8):680-685. doi:10.1111/cns.12713

2. Liu Z, Ren S, Fu K, et al. Restoration of motor function after operative reconstruction of the acutely transected spinal cord in the canine model. Surgery. 2018;163(5):976-983. doi:10.1016/j.surg.2017.10.015.

3. Ren S, Liu Z, Kim CY, et al. Reconstruction of the spinal cord of spinal transected dogs with polyethylene glycol. Surg Neurol Int. 2019;10:50. doi:10.25259/SNI-73-2019.

4. Song RB, Basso DM, da Costa RC, Fisher LC, Mo X, Moore SA. Adaptation of the Basso-Beattie-Bresnahan locomotor rating scale for use in a clinical model of spinal cord injury in dogs. J Neurosci Methods. 2016;268:117-124. doi:10.1016/j.jneumeth.2016.04.023.

5. Ren S, Zhang W, Liu H, et al. Transplantation of a vascularized pedicle of hemisected spinal cord to establish spinal cord continuity after removal of a segment of the thoracic spinal cord: A proof‐of‐principle study in dogs. CNS Neurosci Ther. 2021;27(10):1182-1197. doi:10.1111/cns.13696.

6. Palacios EM, Owen JP, Yuh EL, et al. The evolution of white matter microstructural changes after mild traumatic brain injury: A longitudinal DTI and NODDI study. Sci Adv. 2020;6(32):eaaz6892. doi:10.1126/sciadv.aaz6892.

7. D'Souza M M, Choudhary A, Poonia M, Kumar P, Khushu S. Diffusion tensor MR imaging in spinal cord injury. Injury. 2017;48(4):880-884. doi:10.1016/j.injury.2017.02.016.

8. Cauley KA, Filippi CG. Diffusion-tensor imaging of small nerve bundles: cranial nerves, peripheral nerves, distal spinal cord, and lumbar nerve roots--clinical applications. AJR Am J Roentgenol. 2013;201(2):W326-W335. doi:10.2214/AJR.12.9230.

9. Lu X, Perera TH, Aria AB, Callahan LAS. Polyethylene glycol in spinal cord injury repair: a critical review. J Exp Pharmacol. 2018;10:37-49. doi:10.2147/JEP.S148944.

10. Pasut G, Panisello A, Folch-Puy E, et al. Polyethylene glycols: An effective strategy for limiting liver ischemia reperfusion injury. World J Gastroenterol. 2016;22(28):6501-6508. doi:10.3748/wjg.v22.i28.6501.

Table S1. cBBB scores of beagles at 180 days postoperatively

| cBBB scores | | | | | | | | | | | | |
| --- | --- | --- | --- | --- | --- | --- | --- | --- | --- | --- | --- | --- |
| Weeks | PEG group | | | | Median | Control group | | | | Median | U value | P value |
|  | P1 | P2 | P3 | P4 |  | C1 | C2 | C3 | C4 |  |  |  |
| −1 | 19 | 19 | 19 | 19 | 19 | 19 | 19 | 19 | 19 | 19 | - | - |
| 0 | 0 | 0 | 0 | 0 | 0 | 0 | 0 | 0 | 0 | 0 | - | - |
| 0.5 | 0 | 0 | 0 | 0 | 0 | 0 | 0 | 0 | 0 | 0 | - | - |
| 1 | 0 | 0 | 0 | 0 | 0 | 0 | 0 | 0 | 0 | 0 | - | - |
| 2 | 0 | 0 | 1 | 0 | 0 | 0 | 0 | 0 | 0 | 0 | 6 | 0.686 |
| 3 | 0 | 0 | 3 | 4 | 1.5 | 0 | 0 | 1 | 0 | 0 | 5 | 0.486 |
| 4 | 4 | 4 | 5 | 6 | 4.5 | 0 | 0 | 1 | 1 | 0.5 | 0 | 0.029 |
| 6 | 9 | 8 | 9 | 9 | 9 | 2 | 2 | 2 | 1 | 2 | 0 | 0.029 |
| 8 | 12 | 9 | 10 | 12 | 11 | 2 | 3 | 3 | 2 | 2.5 | 0 | 0.029 |
| 12 | 12 | 10 | 10 | 12 | 11 | 2 | 3 | 3 | 2 | 2.5 | 0 | 0.029 |
| 16 | 12 | 11 | 10 | 12 | 11.5 | 3 | 3 | 4 | 2 | 3 | 0 | 0.029 |
| 20 | 12 | 11 | 10 | 12 | 11.5 | 4 | 4 | 5 | 3 | 4 | 0 | 0.029 |
| 24 | 12 | 11 | 10 | 12 | 11.5 | 4 | 4 | 5 | 3 | 4 | 0 | 0.029 |

cBBB: canine Basso-Beattie-Bresnahan.

Fig. SI. The recovery of hind limb motor function in the PEG and control groups 24 weeks after the surgery. cBBB scores were used to evaluate the hind limb motor function of the beagles. The difference in cBBB scores between the two groups was statistically significant from the fourth week after the surgery (*P* < 0.05).

Fig. S2. Diffusion tensor imaging (DTI) of beagles in PEG and control groups at two weeks postoperatively. The nerve fiber tracts of proximal and distal spinal cords in PEG group have been connected across the operative area (A, B). In the control group, there was a gap between the proximal and distal spinal nerve fibers. The continuity of the spinal cord was not restored (C, D). The color of fiber is only to distinguish the proximal and distal spinal cord tissues, with no other practical significance. A and C show the entire nerve fibers of the spinal cord that are tracked under the DTI. B and D only show the nerve fibers in the operation area that are tracked under the DTI.

Fig. S3. Immunohistochemical staining of the PEG and control groups and the positive staining density of NF-200 and MBP. The NF-200-positive axons and MBP-positive myelin sheaths were observed in the vascularized transplanted spinal cord of PEG group (arrows in A, C), while almost absent in the control group (B, D). The positive staining densities of NF-200 and MBP in PEG group were significantly higher than those in the control group, with a statistically significant difference (*P* < 0.05) (E, F).
